# Supplementary material for: Association between the 2018 WCRF/AICR and the Low-Risk Lifestyle Scores with Colorectal Cancer Risk in the Predimed Study
Source: J Clin Med. 2020 Apr 23;9(4):1215. doi: 10.3390/jcm9041215 (PMC7230705; doi:10.3390/jcm9041215)
Supplement: Supplementary file 1 [file jcm-09-01215-s001.pdf]

Table S1. Third expert report 2018 WCRF/AICR score operationalization in the PREDIMED cohort

	Recommendation	Goals	Operationalization	Scoring
1	Be a healthy weight	Ensure that body weight during childhood and adolescence projects towards the lower end of the healthy adult BMI range	Insufficient data available	-
		Keep your weight as low as you can within the healthy range throughout life	≥18.5 and ≤24.9 Kg/m^2	1
			>24.9 and <30 Kg/m^2	0.5
			<18.5 or ≥30 Kg/m^2	0
		Avoid weight gains (measured as body weight or waist circumference) throughout adulthood[a]	1st tertile of weight change in all the cohort	1
			2nd tertile of weight change in all the cohort	0.5
			3rd tertile of weight change in all the cohort	0
2	Be physically active	Be at least moderately physically active, and follow or exceed national guidelines (about 60 to 75% of heart rate maximum)	≥150 min MVPA/wk.	1
			≥75 and <150 min MVPA/wk.	0.5
			<75 min MVPA/wk.	0
		Limit sedentary habits	Insufficient data available	-
3	Eat a diet rich in wholegrains, vegetables, fruit and beans	Consume a diet that provides at least 30 g/day of fiber from foods	≥30 g/day	1
			≥15 and <30 g/day	0.5
			<15 g/day	0
		Include in most meals foods containing wholegrains, non-starchy vegetables, fruit and pulses (legumes) such as beans and lentils[b]	<1 serv. legumes/wk.	0
			≥1 and <3 serv. legumes/wk.	0.5
			≥3 serv. legumes/wk.	1
		Eat a diet high in all types of plant foods including at least five portions or servings (at least 400 g in total) of a variety of non-starchy vegetables and fruit every day	≥400 g/day	1
			≥200 and <400 g/day	0.5
			<200 g/day	0

	Recommendation	Goals	Operationalization	Scoring
		If you eat starchy roots and tubers as staple foods, eat non-starchy vegetables, fruit and pulses (legumes) regularly too if possible	Insufficient data available	-
4	Limit consumption of 'fast foods' and other processed foods high in fat, starches or sugars	Limit consumption of processed foods high in fat, starches or sugars -including 'fast foods'; many pre-prepared dishes, snacks, bakery foods and desserts; and confectionery (candy)[c]	<1.5 serv./day	1
			≥1.5 and <3 serv./day	0.5
			≥3 serv./day	0
5	Limit consumption of red and processed meat	If you eat red meat, limit consumption to no more than about three portions per week. Three portions are equivalent to about 350 to 500 g cooked weight of red meat. Consume very little, if any, processed meat[d]	RM <450 g/wk. and PM <3 g/day	1
			RM <450 g/wk. and PM ≥3 and <50 g/day	0.5
			RM ≥450 g/wk. and PM ≥50 g/day	0
6	Limit consumption of sugar sweetened drinks	Do not consume sugar-sweetened drinks[d]	0 g/day	1
			>0 g/day and <250 g/day	0.5
			≥250 g/day	0
7	Limit alcohol consumption	For cancer prevention, it is best not to drink alcohol[e]	0 g ethanol/day	1
			>0 to 20 g ethanol/day (women)	0.5
			>0 to 40 g ethanol/day (men)	0.5
			>20 g ethanol/day (women)	0
			>40 g ethanol/day (men)	0
8	Do not use supplements for cancer prevention	High-dose dietary supplements are not recommended for cancer prevention - aim to meet nutritional needs through diet alone	Insufficient data available	-
9	For mothers: breastfeed your baby, if you can	This recommendation aligns with the advice of the WHO, which recommends infants are exclusively breastfed for 6 months, and then up to 2 y of age or beyond alongside appropriate complementary foods	Insufficient data available	-

	Recommendation	Goals	Operationalization	Scoring
10	After cancer diagnosis: follow our Recommendations, if you can	All cancer survivors should receive nutritional care and guidance on physical activity from trained professionals	Insufficient data available	-
		Unless otherwise advised, and if you can, all cancer survivors are advised to follow the Cancer Prevention Recommendations as far as possible after the acute stage of treatment	Insufficient data available	-

[a] Distribution of weight gain in the study population over the follow-up. The mean weight change ± SD (*n*) for the 1st tertile was -5.1 ± 3.7 Kg (2,409 individuals), -0.2 ± 0.7 Kg (2,376 individuals) for the 2nd tertile, and +4.2 ± 3.3 Kg (2,303 individuals) for the 3rd tertile.

[b] The 3rd expert report did not provide cutoffs. Because we have insufficient data available on whole grains consumption, we did not include this food group in the score. Moreover, since fruit and vegetable consumption are considered in another score subcomponent, this item is only composed of legumes consumption (1 serv. = 70 g).

[c] We did not include food groups overlapping with other score components such as processed meats and sugar-sweetened beverages. We included those foods categorized in the 3rd (processed foods) and 4th (ultra-processed foods) groups of the NOVA (a name, not an acronym) classification [1].

[d] The 3rd Expert Report does not provide cut-offs neither for processed meat consumption nor sugar-sweetened beverages intake. Thus, we decided to follow the cutoffs from the previous literature for the comparability of data [2].

[e] The WCRF/AICR recommendations advise not to consume alcoholic drinks. For those individuals consuming alcohol, the recommendations suggest not to exceed the national guidelines. Therefore, we considered the Dietary Guidelines for the Spanish population (SENC, 2016) [3].

Abbreviations: WCRF/AICR, World Cancer Research Fund/American Institute for Cancer Research; BMI, Body Mass Index; MVPA, moderate-to-vigorous physical activity; wk., week; serv., servings; RM, red meat; PM, processed meat; WHO, World Health Organization; SD, standard deviation; SENC, 'Sociedad Española de Nutrición Comunitaria'.

Table S2. Low-risk lifestyle score operationalization in the PREDIMED cohort

	Individual lifestyle risk factors	Operationalization	Scoring
**1**	Smoking status	Never smoking	**1**
**2**	Alcohol consumption	5 to 15 g/day (women) and 5 to 30 g/day (men)	**1**
**3**	Physical activity	≥30 min./day MVPA	**1**
**4**	AHEI-2010	Upper 40% AHEI-2010	**1**
**5**	BMI	BMI (18.5 to 24.9 kg/m^2)	**1**

Abbreviations: min., minutes; MVPA, moderate-to-vigorous physical activity; AHEI, Alternate Healthy Eating Index; BMI, Body Mass Index.

Table S3. Baseline characteristics of the study population in the PREDIMED study according to quartiles of WCRF/AICR score [a].

	Quartiles of 2018 WCRF/AICR score				
	**Q1**	**Q2**	**Q3**	**Q4**	***P*-value[b]**
Events/non-events (*n*)	30/1,868	20/1,793	32/1,911	15/1,644	
2018 WCRF/AICR score	2.9 [2.6-3.2]	3.6 [3.4-3.7]	4.2 [4-4.3]	4.9 [4.7-5.2]	<0.001*
Low-risk lifestyle score	1.5 [1-2]	2 [1-2.5]	2 [1.5-2.5]	2.5 [2-3]	<0.001*
Age, years	66 [62-71]	66 [62-71]	67 [63-72]	67 [63-72]	0.787
Women, % (*n*)	55.5 (1,036)	57.3 (1,027)	58.6 (1,119)	58.6 (963)	0.183
Education level, % (*n*)					
Primary, secondary, high school	91.8 (1,715)	92.1 (1,652)	94.3 (1,802)	93.1 (1,531)	0.014*
University/graduate	8.2 (153)	7.9 (141)	5.7 (109)	6.9 (113)	
Age at diagnosis of cancer, years	73 [68.4-78]	69 [64.7-76.1]	72.4 [67.7-76.5]	71.7 [63.2-74.8]	0.957
Family history of cancer, % (*n*)	49.8 (915)	49 (872)	49.6 (938)	50.5 (823)	0.051
Diabetes, % (*n*)	43.3 (809)	46.5 (833)	50.8 (971)	55.6 (914)	<0.001*
Hypertension, % (*n*)	84.6 (1,581)	83.5 (1,497)	82.6 (1,579)	79.9 (1,313)	0.002*
Waist circumference, cm					
Women	100 [94-108]	100 [92-106]	97 [90-104]	96 [89-103]	0.018*
Men	106 [99-112]	104 [98-110]	101 [96-107]	96 [89-103]	0.179
BMI, kg/m^2	30.8 [28.3-33.4]	30.4 [27.7-32.9]	28.9 [26.8-31.6]	28.7 [26.4-31.3]	<0.001*
MVPA, min./wk.	0 [0-37.8]	18.7 [0-182]	98.4 [0-378]	280 [113.7-507.8]	<0.001*
Smoking status, % (*n*)					
Never smokers	57.2 (1,069)	59.7 (1,070)	64.3 (1,229)	65.1 (1,070)	
Former smokers	25.5 (477)	24.3 (436)	24.1 (460)	24.4 (401)	<0.001*
Current smokers	17.2 (322)	16 (287)	11.6 (222)	10.5 (173)	
Current medication, % (*n*)					
Aspirin	22.9 (427)	22.7 (407)	21.3 (406)	22.7 (373)	0.664
HRT (only in women)	3.7 (38)	2.8 (29)	2.9 (32)	1.7 (16)	0.165
Intervention groups, % (*n*)					
MedDiet+EVOO	33.6 (628)	33.7 (604)	32.9 (629)	37.3 (613)	
MedDiet+nuts	32.6 (609)	33 (592)	34.3 (656)	30.6 (503)	0.107
Control low-fat diet	33.8 (631)	33.3 (597)	32.8 (626)	32.1 (528)	
Energy intake (kcal/day)	2,423 [2,043.1-2,834.8]	2,244 [1,868-2,622]	2,116 [1,800-2,453]	1,995.8 [1,705.4-2,321.5]	<0.001*

AHEI-2010 score	60.3 [54.6-65.7]	63.8 [58.6-69.5]	65.3 [60.6-70.7]	68 [62.8-73.1]	<0.001*
Food consumption, g/day					
Vegetables	294.3 [216.5-383.7]	316.2 [238.3-409.3]	313.7 [237.2-404.3]	336.2 [251.8-433.7]	<0.001*
Fruits	321.4 [213.2-458.1]	332.1 [221.9-482.4]	331.4 [230.7-466.9]	359.7 [243.3-499]	<0.001*
Legumes	16.6 [12-25.1]	17.1 [12.6-25.1]	17.1 [12.6-25.1]	17.1 [12.6-25.7]	<0.001*
Red and processed meat	89.1 [55.7-117.7]	69.1 [43.4-101.4]	62.5 [41.3-93]	54 [32.5-78.4]	<0.001*
Fast food and processed foods	102.6 [72.5-146.4]	77.5 [50-113.9]	65.3 [40.1-94.4]	51.8 [31.4-72.7]	<0.001*
Sugar sweetened beverages	41 [13.3-171.4]	26.7 [0-85.7]	0 [0-85.7]	0 [0-13.3]	<0.001*
Alcohol	4.5 [0.7-13.8]	1.93 [0-11]	1.4 [0-10.4]	0 [0-5.1]	<0.001*

[a] Data are expressed as medians [IQR, interquartile range] for continuous variables and percentage and number (n) for categorical variables. [b] *P*-values for comparison between colorectal cancer cases and non-cases were calculated by chi-square or t-Student tests for categorical and continuous variables, respectively. All statistical tests were two-sided. **P*-value <0.05. Abbreviations: AHEI, Alternate Healthy Eating Index; BMI, body mass index; EVOO, extra virgin olive oil; HRT, hormone replacement therapy; MedDiet, Mediterranean Diet; min./wk., minutes/week; MVPA, moderate-to-vigorous physical activity; WCRF/AICR, World Cancer Research Fund/American Institute for Cancer Research.

Table S4. Baseline characteristics of the study population in the PREDIMED study according to tertiles of low-risk lifestyle score [a].

	Tertiles of low-risk lifestyle score			
	**T1**	**T2**	**T3**	***P*-value[b]**
Events/non-events (*n*)	55/3,024	27/2,512	15/1,583	
Low-risk lifestyle score	1 [1-1.5]	2 [2-2.5]	3.5 [3-3.5]	<0.001*
2018 WCRF/AICR score	3.5 [3-4]	3.9 [3.4-4.4]	4.4 [3.8-4.9]	0.790
Age, years	67 [62-72]	67 [62-72]	67 [63-72]	0.154
Women, % (*n*)	54.8 (1,688)	62 (1,574)	55.3 (883)	<0.001*
Education level, % (*n*)				
Primary, secondary, high school	92.5 (2,848)	93.7 (2,380)	92.1 (1,472)	0.087
University/graduate	7.5 (231)	6.3 (159)	7.9 (126)	
Age at diagnosis of cancer, years	72.6 [68-76.3]	68.4 [65.1-75.9]	73.5 [64.5-78]	0.154
Family history of cancer, % (*n*)	50.2 (1,527)	48.2 (1,209)	51.1 (812)	<0.001*
Diabetes, % (*n*)	49.4 (1,520)	48.3 (1,225)	48.9 (782)	0.704
Hypertension, % (*n*)	83.1 (2,558)	83.5 (2,121)	80.8 (1,291)	0.060
Waist circumference, cm				
Women	100 [93-107]	98 [91-106]	95 [87-102]	0.023*
Men	104 [99-111]	102 [97-108]	100 [95-107]	0.158
BMI, kg/m^2	30.3 [27.9-33]	29.7 [27.3-32.5]	28.4 [25.8-31.3]	<0.001*
MVPA, min./wk.	0 [0-74.8]	70.1 [0-342.8]	303.4 [149.5-556]	<0.001*
Smoking status, % (*n*)				
Never smokers	47.9 (1,476)	68.5 (1,738)	76.6 (1,224)	
Former smokers	31.7 (975)	21.1 (535)	16.5 (264)	<0.001*
Current smokers	20.4 (628)	10.5 (266)	6.9 (110)	
Current medication, % (*n*)				
Aspirin	22.5 (694)	22.1 (562)	22.3 (357)	0.709
HRT (only in women)	3.4 (57)	2.4 (38)	2.3 (20)	0.329
Intervention groups, % (*n*)				
MedDiet+EVOO	33.7 (1,037)	35.3 (895)	33.9 (542)	
MedDiet+nuts	30.7 (945)	31.8 (808)	38 (607)	<0.001*
Control low-fat diet	35.6 (1,097)	32.9 (836)	38.1 (449)	
Energy intake (kcal/day)	2,227 [1,854.5-2,653.3]	2,135 [1,811.8-2,514.7]	2,186 [1,875.7-2,530.4]	<0.001*
AHEI-2010 score	60.4 [55.6-63.9]	66.5 [60.7-71.4]	70.8 [67.5-75.1]	<0.001*

Food consumption, g/day				
Vegetables	293.7 [218-380]	318.7 [244.8-419.7]	347.1 [259.8-445]	<0.001*
Fruits	300 [198.3-425]	349.2 [242.6-493.3]	379.5 [268.3-527.9]	<0.057
Legumes	16.6 [12-25.1]	20.6 [16-25.1]	20.6 [16-25.1]	<0.001*
Red and processed meat	75.7 [47.1-106.2]	63.8 [39.9-97.6]	60.5 [38.2-91.6]	<0.001*
Fast food and processed foods	76.4 [48.2-114.8]	71.2 [44.3-106]	68 [44.2-101.8]	<0.001*
Sugar sweetened beverages	26.7 [0-114.3]	13.3 [0-85.7]	0 [0-41.9]	<0.001*
Alcohol	0.7 [0-4.5]	1.5 [0-10.4]	10.2 [1.4-12.5]	<0.001*

[a] Data are expressed as medians [IQR, interquartile range] for continuous variables and percentage and number (n) for categorical variables. [b] *P*-values for comparison between colorectal cancer cases and non-cases were calculated by chi-square or t-Student tests for categorical and continuous variables, respectively. All statistical tests were two-sided. **P*-value <0.05. Abbreviations: AHEI, Alternate Healthy Eating Index; BMI, body mass index; EVOO, extra virgin olive oil; HRT, hormone replacement therapy; MedDiet, Mediterranean Diet; min./wk., minutes/week; MVPA, moderate-to-vigorous physical activity; WCRF/AICR, World Cancer Research Fund/American Institute for Cancer Research.

Table S5. Mutually adjusted HRs and 95% CIs for colorectal cancer risk associated with different categories of a combination of low-risk lifestyle factors at baseline in the PREDIMED study (*n*=7,216)

	**Number of low-risk factors, HR (95% CI)**					**P for trend**
	**0 (reference)**	**1**	**2**	**>3**	**Continuous analysis (1-point increment)**	
Events/non-events (*n*)	9/601	46/2,423	27/2,512	15/1,583		
Crude model	1.00	1.31 (0.62-2.79)	0.72 (0.32-1.65)	0.61 (0.24-1.52)	0.75 (0.60-0.94)*	0.013*
Model 1	1.00	1.58 (0.75-3.34)	0.87 (0.39-1.96)	0.70 (0.28-1.73)	0.77 (0.62-0.96)*	0.018*
Model 2	1.00	1.59 (0.76-3.34)	0.86 (0.38-1.94)	0.69 (0.28-1.70)	0.77 (0.62-0.95)*	0.016*

Model 1 adjusted for age (years, continuous) and sex.

Model 2 was further adjusted for intervention group (MedDiet + EVOO, MedDiet + nuts, low-fat control), family history of cancer (yes/no), education level (primary or secondary/high school university or graduate), history of diabetes (yes/no), baseline energy intake (Kcal/day, continuous) and treatment with aspirin (yes/no) at baseline.

*p-value <0.05

Abbreviations: HR, Hazard Ratio; CI, confidence interval; MedDiet, Mediterranean Diet; EVOO, extra virgin olive oil.

Table S6. HRs and 95% CIs between the 2018 WCRF/AICR and the low-risk lifestyle scores and colorectal cancer risk at baseline in the PREDIMED study by subgroups (age, sex and T2D status)

	**2018 WCRF/AICR score**		**Low-risk lifestyle score**	
	**Continuous analysis (1-point increment), HR (95% CI)**	***P* for trend**	**Continuous analysis (1-point increment), HR (95% CI)**	***P* for trend**
**Age< 67 years**				
Events/non-events (*n*)	41/3,499	-	41/3,499	-
Crude model	0.89 (0.68-1.17)	0.403	0.78 (0.59-1.05)	0.099
Model 1	0.90 (0.68-1.19)	0.458	0.79 (0.60-1.05)	0.110
Model 2	0.88 (0.67-1.17)	0.382	0.79 (0.60-1.05)	0.109
**Age≥ 67 years**				
Events/non-events (*n*)	56/3,717	-	56/3,717	-
Crude model	0.70 (0.50-0.97)*	0.032*	0.73 (0.54-0.99)*	0.041*
Model 1	0.70 (0.50-0.97)*	0.032*	0.76 (0.58-1.00)	0.051
Model 2	0.71 (0.50-0.99)*	0.050*	0.76 (0.58-0.99)*	0.048*
**Men**				
Events/non-events (*n*)	57/3,071	-	57/3,071	-
Crude model	0.86 (0.64-1.16)	0.323	0.85 (0.66-1.10)	0.213
Model 1	0.80 (0.58-1.11)	0.185	0.83 (0.65-1.07)	0.154
Model 2	0.85 (0.62-1.17)	0.315	0.84 (0.65-1.08)	0.174
**Women**				
Events/non-events (*n*)	40/4,145	-	40/4,145	-
Crude model	0.75 (0.55-1.01)	0.059	0.69 (0.50-0.96)*	0.028*
Model 1	0.75 (0.55-1.02)	0.064	0.70 (0.50-0.97)*	0.030*
Model 2	0.72 (0.51-1.02)	0.064	0.69 (0.50-0.95)*	0.023*

**Prevalent T2D**				
Events/non-events (*n*)	50/3,527	-	50/3,527	-
Crude model	0.69 (0.51-0.93)*	0.014*	0.81 (0.60-1.07)	0.142
Model 1	0.68 (0.50-0.92)*	0.012*	0.81 (0.62-1.07)	0.138
Model 2	0.71 (0.53-0.96)*	0.024*	0.81 (0.62-1.06)	0.123
**Non-prevalent T2D**				
Events/non-events (*n*)	47/3,689	-	47/3,689	-
Crude model	0.89 (0.64-1.23)	0.475	0.69 (0.50-0.95)*	0.021*
Model 1	0.90 (0.64-1.27)	0.550	0.72 (0.53-0.97)*	0.028*
Model 2	0.87 (0.61-1.24)	0.443	0.71 (0.52-0.95)*	0.021*

Model 1 adjusted for age (years, continuous) and sex.
Model 2 was model 1 plus intervention group (MedDiet + EVOO, MedDiet + nuts, low-fat control), family history of cancer (yes/no), education level (primary or secondary/high school university or graduate), history of diabetes (yes/no), baseline energy intake (Kcal/day, continuous) and treatment with aspirin (yes/no) at baseline. Model 2 for 2018 WCRF/AICR score was further adjusted for current smoker (yes/no), former smoker (yes/no), never smoker (yes/no).
All models were stratified by node. *p-value <0.05
Abbreviations: HR, Hazard Ratio; CI, confidence interval; WCRF/AICR, World Cancer Research Fund/American Institute for Cancer Research; T2D, type 2 diabetes; MedDiet, Mediterranean Diet; EVOO, extra virgin olive oil.

References

1. Monteiro, C.A.; Cannon, G.; Moubarac, J.-C.; Levy, R.B.; Louzada, M.L.C.; Jaime, P.C. The UN Decade of Nutrition, the NOVA food classification and the trouble with ultra-processing. *Public Health Nutr.* **2018**, *21*, 5–17.
2. Romaguera, D.; Vergnaud, A.-C.; Peeters, P.H.; van Gils, C.H.; Chan, D.S.M.; Ferrari, P.; Romieu, I.; Jenab, M.; Slimani, N.; Clavel-Chapelon, F.; et al. Is concordance with World Cancer Research Fund/American Institute for Cancer Research guidelines for cancer prevention related to subsequent risk of cancer? Results from the EPIC study. *Am. J. Clin. Nutr.* **2012**, *96*, 150–63.
3. Aranceta Bartrina, J.; Aranceta Bartrina, J.; Arija Val, V.; Maíz Aldalur, E.; Martínez de la Victoria Muñoz, E.; Ortega Anta, R.M.; Pérez-Rodrigo, C.; Quiles Izquierdo, J.; Rodríguez Martín, A.; Román Viñas, B.; et al. Guías alimentarias para la población española (SENC, 2016); la nueva pirámide de la alimentación saludable. *Nutr. Hosp.* **2016**, *33*, 1–48.
